# Resting-state brain function and its modulation by intranasal oxytocin in antisocial personality disorder with and without psychopathy

**DOI:** 10.1038/s41598-026-36661-5

**Published:** 2026-01-25

**Authors:** Julia Griem, Daniel Martins, John Tully, Declan Murphy, Yannis Paloyelis, Nigel Blackwood

**Affiliations:** 1 Department of Forensic and Neurodevelopmental Sciences, Institute of Psychiatry, Psychology and Neuroscience, King’s College London, 16 De Crespigny Park, London, SE5 8AB UK; 2 Department of Neuroimaging Institute of Psychiatry, Psychology and Neuroscience, King’s College London, Centre for Neuroimaging Sciences, De Crespigny Park, London, SE5 8AB UK; 3 Academic Unit of Mental Health and Clinical Neurosciences, School of Medicine, Institute of Mental Health, University of Nottingham, University of Nottingham Innovation Park, Triumph Road, Nottingham, NG7 2TU UK

**Keywords:** Antisocial personality disorder, Psychopathy, Oxytocin, Perfusion, RCBF, Resting-state brain function, Neuroscience, Psychology

## Abstract

Behavioural, structural, and functional neuroimaging differences exist between individuals with antisocial personality disorder with (ASPD + P) or without psychopathy (ASPD-P). However, the aetiological mechanisms underpinning such differences remain unclear, hindering treatment development. Intranasal oxytocin (OT) has shown modulatory effects on social brain function in healthy and antisocial populations. We investigated the effects of OT on resting-state brain function in individuals with violent offending histories with ASPD+/-P using arterial spin labelling to measure regional cerebral blood flow (rCBF). A double-blind, placebo-controlled, crossover design was employed with males with ASPD (ASPD + P: *N* = 17, ASPD-P: *N* = 14) and healthy male non-offenders (*N* = 22). Both ASPD subtypes exhibited reduced rCBF in frontotemporal regions compared to non-offenders. Individuals with ASPD + P showed significantly greater rCBF increases in posterior default mode network regions compared to individuals with ASPD-P. OT administration selectively decreased rCBF in the left basal ganglia of the ASPD-P group, an effect not observed in ASPD + P or non-offender groups. These findings highlight functional brain differences between individuals with ASPD + P and ASPD-P at rest and demonstrate oxytocin’s differential impact on resting-state measures. Further understanding of the origins of these neurobiological differences could inform targeted therapeutic strategies for individuals with ASPD with and without psychopathy.

**Supplementary Information:**

The online version contains supplementary material available at 10.1038/s41598-026-36661-5.

## Introduction

Antisocial personality disorder (ASPD) is characterized by impulsivity, irresponsibility and aggression^1^. Approximately one third of individuals with ASPD additionally meet categorical criteria for psychopathy (ASPD+P)^2^, characterized by callous unemotional behaviours and a lack of remorse^3^. Antisocial individuals with (ASPD+P) and without (ASPD-P) psychopathy share features such as life-course persistent offending and heightened reactive aggression^4,5^. Those individuals with ASPD+P demonstrate an earlier onset and greater density of offending behaviours, increased use of proactive aggression, and a poorer response to treatment interventions than those with ASPD-P^6–10^.

Behavioural similarities between the two antisocial subgroups are underpinned by convergent neurobiological abnormalities. Meta-analytic evidence indicates that functional abnormalities in the orbitofrontal cortex, anterior cingulate, amygdala, and insula are observed in men with ASPD undertaking social and affective cognitive tasks^11^. Imbalances in the striatal excitation/inhibition ratio, which may underpin shared problems with impulsive decision-making^12^, have been observed in those with ASPD+P and ASPD-P in comparison to healthy individuals^13^. A small number of studies also suggest that the behavioural differences between the two groups are underpinned by neurobiological differences. Antisocial men with ASPD+P have reduced grey matter volumes in the anterior prefrontal cortex and the bilateral temporal poles^14^ and abnormal frontotemporal activity during empathic and reward processing tasks^15–17^ in comparison to antisocial men without psychopathy and healthy individuals.

While prior functional neuroimaging (fMRI) studies in these populations have predominantly focused on task-related brain function in ASPD, resting-state fMRI analysis offers unique insights into baseline neural activity independent of specific behavioural demands^18–21^. Resting-state function is linked to, and may even predict, general cognitive propensities that underpin behaviour such as decision-making^22–24^. Neuroimaging studies investigating resting-state brain activity in large samples of incarcerated individuals with varying degrees of psychopathy have suggested abnormalities in the form of aberrant functional connectivity or topology in frontotemporal and limbic regions including the anterior cingulate, insula, and amygdala, as well as in large-scale brain networks including the default mode network (DMN)^25–27^. Two studies of young men meeting criteria for ASPD have also implicated these regions as well as the frontoparietal attention network^28,29^. However, no study to date has directly compared resting-state brain activity between ASPD+P and ASPD-P populations.

In addition, resting-state imaging studies in these populations have typically relied on measurements of the blood-oxygen-level-dependent (BOLD) contrast, a proxy measure of neural activity based on the complex interaction between blood flow, volume and oxygen metabolism^30,31^. A closely related physiological measure, regional cerebral blood flow (rCBF) provides a more direct proxy of neural activity by indexing perfusion from cerebral capillaries into brain tissue. A small number of studies have measured rCBF using single photon emission computed tomography (SPECT) or positron emission tomography (PET) in antisocial populations, typically demonstrating decreased perfusion in anterior and medial (cingulate) frontal regions, lateral temporal regions, the amygdala and insula^32,33^, and increased perfusion in posterior DMN regions^34^. However, these studies were constrained by the recruitment of heterogenous clinical and non-clinical samples, including individuals characterised by aggression, antisocial behaviours or substance misuse disorders, but without detailed phenotypic characterization of ASPD+/-P. Furthermore, these studies used large, manually defined regions of interest and non-automated analytic approaches (e.g., visual judgment) to perfusion data. These limitations can be addressed by using arterial spin labelling (ASL), a non-invasive measure of rCBF with improved spatial resolution and excellent test-retest reliability^35,36^, to assess resting-state rCBF in individuals with carefully phenotyped ASPD+/-P.

Measurement of resting-state brain activity in clinical populations also offers a context-free measure of the brain’s responsivity to pharmacological modulation. One pharmacological agent of particular interest to the ASPD+/-P populations is oxytocin, a neuropeptide central to the regulation of complex social behaviours such as empathy^37,38^. Two recent task-based fMRI studies revealed that a single dose of intranasal oxytocin (OT) normalised aberrant neural responsivity to fearful faces in the anterior cingulate and anterior insula in the ASPD+P (but not ASPD-P) group^17^ and angry faces in the amygdala in a broadly defined ASPD group^39^. However, whether OT also modulates resting-state brain function in ASPD with or without psychopathy remains unknown. Gaining an understanding of the effect of OT on resting-state brain function in ASPD would provide valuable insight into the ‘shiftability’ (i.e., the responsivity to pharmacological modulation) of baseline brain dysfunction in the disorder^40^.

ASL imaging is more sensitive to the effects of pharmacological challenges than BOLD imaging because rCBF is less susceptible to non-specific drug effects than the BOLD signal^31,41–43^. ASL may thus be preferred for investigating OT effects on resting-state brain function. Its ability to capture the time-, method-, and dose-dependent changes in rCBF in response to oxytocin has been demonstrated^44^. In healthy individuals, OT modulates rCBF in regions including the amygdala and the anterior insula^44–46^. Importantly, these are areas where dysregulated neural activity has been demonstrated in ASPD+/-P populations^15,16^ and where task-related brain activity in individuals with ASPD+P was modulated with OT^17,39^.

We thus employed ASL imaging to examine potential differences in resting-state brain function and the impact of a single acute intranasal administration of OT on such resting-state rCBF in males with a history of violent offending with ASPD+P (*N* = 17) and ASPD-P (*N* = 22) and healthy male non-offenders (*N* = 22) using a double-blind, placebo-controlled, randomised crossover design. We conducted region-of-interest (ROIs: amygdala/anterior insula) and exploratory whole-brain analyses. The ROIs were selected a priori due to consistent evidence of structural and functional abnormalities in these areas in the disorder^11,47,48^ together with their demonstrated responsivity to OT in functional imaging paradigms^17,39^. For the ROI analysis, we hypothesized that: (1) both ASPD groups would show reduced amygdala and anterior insula rCBF in comparison to the non-offender group; and (2) such differences would be more marked in the ASPD+P group in comparison to the ASPD-P group. Considering the distinct normalising effects of OT on fearful face processing in ASPD+P^17^, we further hypothesized that (3) OT would restore rCBF in these ROIs in individuals with ASPD+P but not ASPD-P. For the whole-brain analyses, we further expected to find that (4) both ASPD groups would show reduced anterior frontal, anterior cingulate and lateral temporal lobe rCBF, together with increased rCBF in posterior DMN regions such as posterior cingulate and precuneus in comparison to the non-offender group and that (5) such differences would be more marked in the ASPD+P group in comparison to the ASPD-P group.

## Results

### Sample characteristics

Table 1 shows the demographic and clinical characteristics of the three participant groups. They did not differ significantly in age or IQ. As expected, the three groups differed significantly in years of education (the ASPD groups had fewer years of education than the non-offender group), PCL-R scores, and the aggression subscales (the ASPD groups scored higher than the non-offender group, and the ASPD+P group scored higher than the ASPD-P group). Also as expected, the ASPD+P group had a higher frequency of comorbid cluster A personality disorders than the ASPD-P group (see supplementary Table 1 for a breakdown of the comorbidities of individual personality disorders). This is in keeping with the normal range of variation in clinical profiles of ASPD+/-P^49^ and we did not adjust our analyses based on these findings. The two ASPD groups did not differ significantly in comorbid substance use disorder prevalence or positive urine drug screening tests on the days of scanning. To avoid over-correcting for phenotypic variance inherent to ASPD^50^, substance use was not included as a covariate in our main analyses. However, we note that the ASPD+P group had a significantly higher rate of cocaine use as compared with the non-offender group. Therefore, we conducted supplementary analyses which included substance use as a covariate (see supplementary Table 4 for the details about drug use across groups and supplementary results for covariate analyses). Global CBF did not differ between groups or treatment conditions (see supplementary Table 2).

**Table 1:** **Demographic and clinical characteristics of participants.** Data are mean (standard deviation) unless otherwise stated. Non-offender participants did not have any diagnosis of personality disorder or mental illness and no history of conviction, therefore only the two ASPD groups were compared on these variables. Some participants did not complete the RPQ (final ASPD+P N = 13, ASPD-P N = 13, NO N = 20). RPQ = reactive proactive aggression questionnaire, PD = personality disorder, SUD = substance use disorder within past 12 months. F-statistic = ANOVA with Tukey post-hoc, H-statistic = Kruskal Wallis with Mann Whitney U post hoc, ^†^Fisher’s exact test with fisher’s exact post-hoc, χ^2^ = chi-squared test of independence with chi-squared test of independence as post hoc, U-statistic = Mann Whitney U. ◊ pairwise comparison did not survive a Sidak correction for multiple comparisons (adjusted α = 1 – (1-0.05.05)^1/3^ = 0.017).

Demographic	ASPD+*P* (*N* = 17)	ASPD-*P* (*N* = 14)	NO (*N* = 22)	Group comparison	Post hoc tests (*p*-values)		
					NO vs. ASDP+P	NO vs. ASPD-P	ASPD+P vs. ASPD-P
Age (years)	40.56 (9.95)	45.21 (10.05)	37.73 (9.56)	H_(2)_ = 4.44, *p* = 0.11	.	.	.
IQ	91.56 (12.45)	99.14 (15.24)	98.95 (10.77)	H_(2)_ = 3.44, *p* = 0.18	.	.	.
Duration of education (years)	9.88 (1.82)	10.64 (2.21)	13.77 (3.27)	H_(2)_ = 19.40, *p* < 0.001	*p* < 0.001	*p* = 0.002	*p* = 0.28
Age at first violent conviction	20.38 (5.45)	21.43 (5.03)	.	t = 0.55, *p* = 0.59	.	.	.
Violent convictions	4.31 (2.87)	3.86 (2.93)	.	U = 112.00, *p* = 0.78	.	.	.
Reconviction within 3 years, N (%)	8 (47%)	6 (43%)	.	0.06, *p* = 1.00 ^†^	.	.	.
RPQ reactive aggression	15.77 (4.69)	12.46 (5.77)	5.90 (3.54)	F_(2)_ = 19.90, *p* < 0.001	*p* < 0.001	*p* < 0.001	*p* = 0.22
RPQ proactive aggression	13.85 (6.39)	7.38 (6.41)	0.75 (1.16)	H_(2)_ = 29.30, *p* < 0.001	*p* < 0.001	*p* < 0.001	*p* = 0.03^ns◊^
RPQ total aggression	29.62 (9.91)	19.85 (11.46)	6.65 (4.03)	H_(2)_ = 27.56, *p* < 0.001	*p* < 0.001	*p* < 0.001	*p* = 0.04^ns◊^
PCL-R Factor 1	9.24 (3.05)	4.81 (3.03)	1.09 (1.63)	H_(2)_ = 35.41, *p* < 0.001	*p* < 0.001	*p* < 0.001	*p* < 0.001
PCL-R Facet 1 (interpersonal)	4.24 (1.82)	1.81 (1.63)	0.64 (0.95)	H_(2)_ = 27.80, *p* < 0.001	*p* < 0.001	*p* = 0.02^ns◊^	*p* < 0.001
PCL-R Facet 2 (affective)	5.00 (1.80)	3.00 (1.80)	0.46 (0.80)	H_(2)_ = 34.47, *p* < 0.001	*p* < 0.001	*p* < 0.001	*p* = 0.01
PCL-R Factor 2	16.17 (1.69)	11.43 (3.08)	1.09 (1.44)	H_(2)_ = 43.40, *p* < 0.001	*p* < 0.001	*p* < 0.001	*p* < 0.001
PCL-R Facet 3 (lifestyle)	7.59 (1.23)	5.36 (1.69)	1.00 (1.23)	H_(2)_ = 41.07, *p* < 0.001	*p* < 0.001	*p* < 0.001	*p* < 0.001
PCL-R Facet 4 (antisocial)	8.47 (1.33)	6.07 (2.13)	0.50 (1.14)	H_(2)_ = 41.84, *p* < 0.001	*p* < 0.001	*p* < 0.001	*p* = 0.002
PCL-R Total	28.21 (3.22)	17.51 (4.55)	2.64 (2.97)	H_(2)_ = 45.55, *p* < 0.001	*p* < 0.001	*p* < 0.001	*p* < 0.001
PD other than ASPD, N (%)							
Cluster A	5 (29%)	0 (0)	.	4.91, *p* = 0.05^†^	.	.	.
Cluster B	8 (47%)	2 (14%)	.	3.77, *p* = 0.07^†^	.	.	.
Cluster C	1 (6%)	2 (14%)	.	0.62, *p* = 0.58^†^	.	.	.
SUD, N (%)	3 (18%)	4 (29%)	.	0.52, *p* = 0.67^†^	.	.	.
ADHD, N (%)	2 (12%)	1 (7%)	.	0.19, *p* = 1.00^†^	.	.	.
Positive urine drug test, N (%)							
Placebo scan	11 (65%)	3 (23%)	5 (23%)	8.13, *p* = 0.02^†^	*p* = 0.01	*p* = 1.00	*p* = 0.03^ns◊^
Oxytocin scan	12 (71%)	5 (36%)	6 (27%)	χ2 = 7.78, *p* = 0.02	*p* = 0.01	*p* = 0.72	*p* = 0.08

### Group, treatment, and interaction effects on rCBF

The a priori ROI analysis in the amygdala and anterior insula did not reveal significant group, treatment, or interaction effects (see supplementary Table 3). The additional Bayesian linear mixed models revealed strong evidence in favour of the null hypothesis (see supplementary materials). This indicated that there is a very low likelihood for group differences or treatment effects (or their interaction) on rCBF in the four ROIs.

**Fig. 1:**
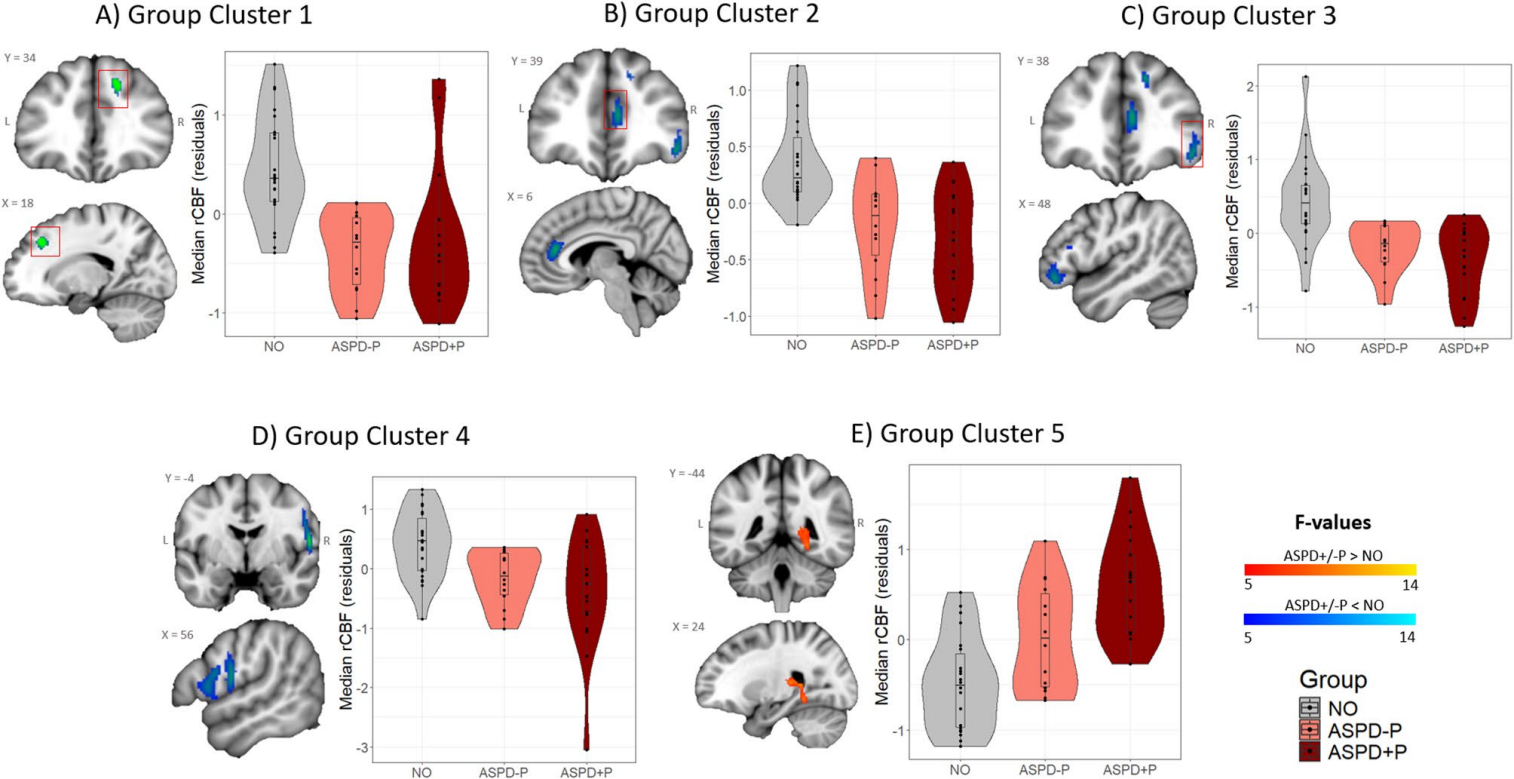
**Clusters with significant group differences in rCBF**. The violin plots (with box plots inside) show the marginal mean and individual datapoints (z-standardized residuals) to depict spread of the median rCBF values within each significant cluster, and to clarify post-hoc what was driving the F-effects identified in the whole-brain analysis. For D), the main effect of group and the pairwise comparisons remained significant (p = 0.008) after removing the ASPD+P outlier (z-standardized residual > |3.0|), so this was kept in the analysis to increase power. Blue shaded clusters indicate reductions, red shaded cluster indicates increase. A red box visually highlights the cluster that the plot is referring to (in case other clusters are also visible in that slice).

**Fig. 2:**
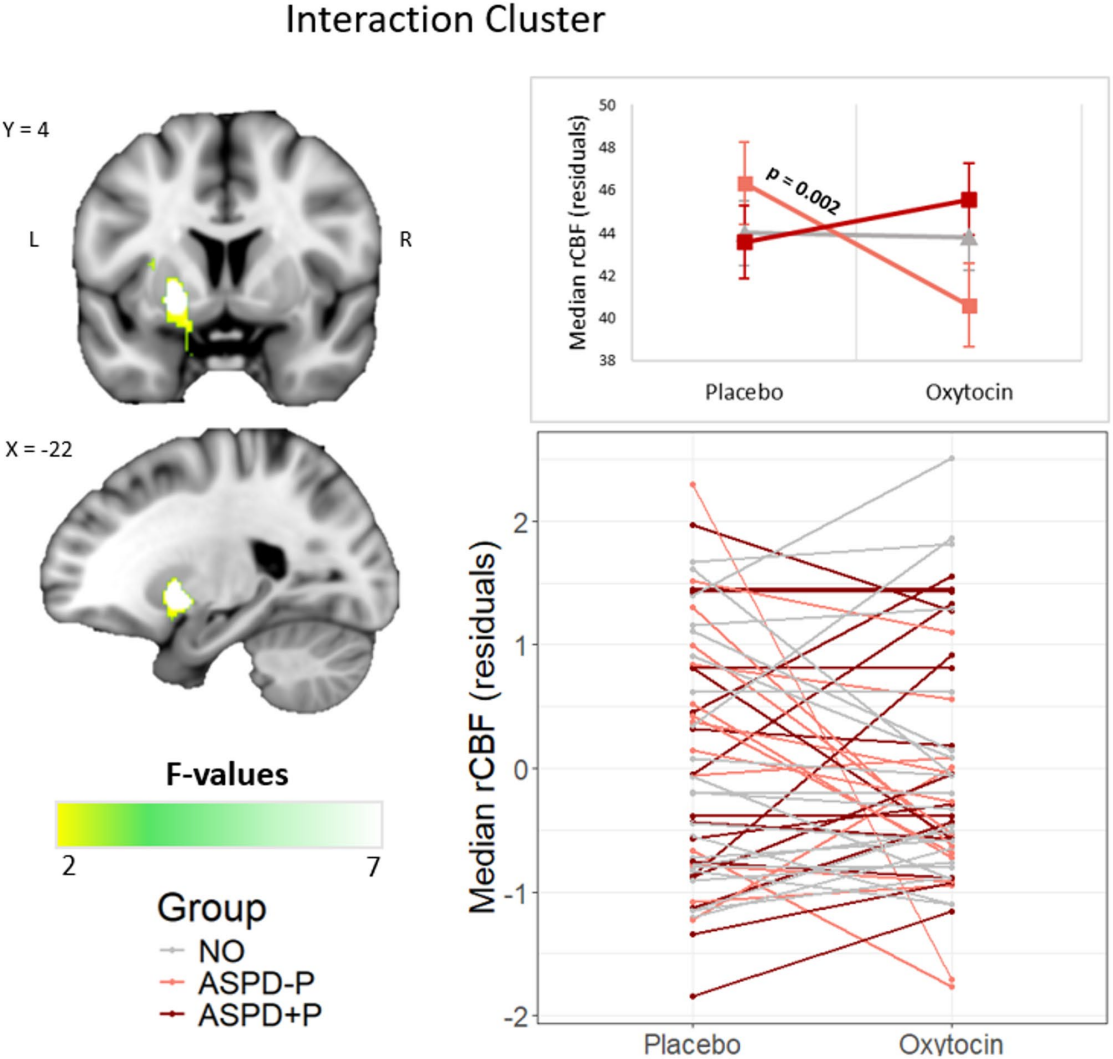
**Cluster with a significant group by treatment interaction effect on rCBF.** The top line plot shows the marginal means (EMMs) of the median rCBF values for each group under each treatment condition, after accounting for the effect of global median CBF, age, and minutes since dose. The bottom spaghetti plot shows individual participants’ responsivity to OT. The interaction effect and simple main effect remain significant (p = 0.01) after excluding the single ASPD-P participant with the steepest slope.

The whole-brain analysis revealed a significant main effect of group in five clusters (Table 2; Fig. 1). Post-hoc pairwise comparisons showed that both ASPD+P and ASPD-P groups had reduced rCBF relative to the non-offender group in four of these clusters. These four clusters spanned the right hemisphere frontal and temporal areas (medial superior frontal gyrus, anterior cingulate cortex, orbitofrontal cortex, Rolandic operculum, pre-/post-central gyrus, and superior temporal gyrus). By contrast, in the fifth cluster, which encompassed core areas of the posterior DMN (right hemisphere posterior cingulate, precuneus, hippocampus), both ASPD groups had increased rCBF relative to the non-offender group, and individuals with ASPD+P had further increased rCBF compared to individuals with ASPD-P. There were no significant main effects of treatment. However, a significant group by treatment interaction effect was found in one cluster spanning the left basal ganglia, specifically the globus pallidus, putamen and caudate (Table 2; Fig. 2). Simple main effects tests revealed that this was driven by a significant decrease in rCBF after OT in the ASPD-P group only. These results were largely unchanged after adding substance use (presence of a positive drug screen) as a covariate (see supplementary materials).

**Table 2:** **Clusters with significant group and interaction effects on rCBF.**

Cluster-wise analysis								Pairwise Comparisons					
Cluster description	Hemisphere	K	*P*_FWE_	F	Peak coordinates			Estimated Marginal Means (SE)			Sidak-corrected *p*-values		
					x	y	z						
Main effect of group (F-contrast)								ASPD+P	ASPD-P	NO	ASPD+P vs. NO	ASPD-P vs. NO	ASPD+P vs. ASPD-P
Cluster 1: Medial superior frontal gyrus	Right	410	< 0.001	18.12	18	34	40	39.74 (0.91)	38.68 (1.03)	44.70 (0.81)	*p* < 0.001	*p* < 0.001	*p* = 0.83
Cluster 2: Anterior cingulate cortex	Right	218	0.007	10.77	6	36	16	57.55 (0.75)	58.48 (0.85)	62.77 (0.66)	*p* < 0.001	*p* < 0.001	*p* = 0.80
Cluster 3: Pars orbitalis, orbitofrontal cortex	Right	163	0.04	10.32	46	40	−12	58.22 (1.07)	59.66 (1.22)	65.53 (0.95)	*p* < 0.001	*p* = 0.002	*p* = 0.77
Cluster 4: Rolandic operculum, pre- and postcentral gyrus, superior temporal gyrus	Right	490	< 0.001	13.26	58	−4	12	49.77 (1.66)	51.75 (1.88)	57.75 (1.47)	*p* = 0.002	*p* = 0.05	*p* = 0.82
Cluster 5: Posterior (isthmus) cingulate cortex, precuneus, hippocampus	Right	270	0.001	9.90	22	−42	8	35.14 (0.71)	32.31 (0.81)	29.49 (0.63)	*p* < 0.001	*p* = 0.03	*p* = 0.04

Group x treatment interaction effect (F-contrast)											PL vs. OT		
											ASPD + P	ASDP-P	NO
Globus pallidus, putamen, caudate	Left	258	0.02	9.32	−22	2	−6	PL: 43.57 (1.78) OT: 45.57 (1.78)	PL: 46.33 (2.05) OT: 40.62 (2.05)	PL: 43.99 (1.62) OT: 43.80 (1.62)	*p* = 0.20	*p* = 0.002	*p* = 0.89

Main effects were measured with F-contrasts. *PL = *placebo scan,* OT = *oxytocin scan. Clusters labelled according to Automated Anatomical Labelling (AAL3) atlas built into SPM12 and confirmed by mapping MNI peak coordinates to Talairach space in BioImage Suite (https://bioimagesuiteweb.github.io/bisweb-manual/tools/mni2tal.html). *SE* = standard error.

## Discussion

This study provides novel evidence that individuals with a history of violent offending with ASPD+P or ASPD-P exhibit shared and distinct resting-state neurobiological features. We did not establish significant differences in resting-state rCBF between groups, or in response to OT, in either the amygdala or the anterior insula (our a priori ROIs; hypotheses 1–3). However, we did reveal the expected reductions in resting-state rCBF in frontotemporal regions in both antisocial groups compared to non-offenders, and the increased rCBF in posterior DMN regions in the ASPD+P group compared to the ASPD-P and non-offender groups (hypotheses 4 and 5). Moreover, OT selectively reduced resting-state rCBF in the left basal ganglia of the ASPD-P group but had no significant effect in the ASPD+P or non-offender groups.

The a priori ROI analysis did not reveal the expected resting-state rCBF abnormalities in the anterior insula or amygdala in the ASPD+/-P groups, supporting a recent suggestion that observed functional impairments in these brain regions in the disorder are task-dependent and context-evoked^51^, i.e., they may not have been detected in our resting-state paradigm. Equally, OT did not exert a significant modulatory effect on resting-state rCBF in these pre-specified ROIs in any group. The latter was surprising given that previous research had demonstrated resting-state rCBF changes in the anterior insula and amygdala in healthy individuals^44–46^. However, differences in the dose and timing of oxytocin administration could explain the discrepant findings. Thus, maximal resting-state effects appear to be associated with lower OT doses such as 9–18 IU^46^. OT effects in the amygdala, insula, or anterior cingulate of healthy individuals have typically been captured at an earlier time point (25–78 min post-administration^45^). However, our study was adequately powered to detect between-group rCBF differences in a crossover design^52^ and the Bayesian linear mixed models provided compelling evidence in favour of the null hypothesis in the context of this OT dose (40 IU) and time frame of post-dose assessment (85 min). Ultimately, confidence in such null findings will be best derived from mega-analytic data from collaborative neuroimaging consortia such as the ENIGMA-Antisocial Behaviour group (https://enigma.ini.usc.edu/ongoing/enigma-antisocial-behavior/). Such consortia benefit from access to within-subject multi-modal data from multiple centres, enabling improved statistical power to reveal true effects.

The exploratory whole-brain analysis did reveal the expected reductions in rCBF in both ASPD+P and ASPD-P groups compared to the non-offender group in predominantly frontal, and to a lesser extent temporal regions. These findings are consistent with (but more spatially precise than) earlier SPECT^33^ and PET^32^ findings in adult antisocial populations. These results (except for the reduced rCBF in the ASPD-P group within cluster 4) remained significant after covarying for the presence of recent substance use (see supplementary materials), suggesting that the demonstrated differences between the antisocial and non-offender groups are not simply attributable to the potential confound of comorbid substance use.

These results could have potential implications for understanding the neurobiological mechanisms underpinning the shared behavioural characteristics of individuals with ASPD+P and ASPD-P. It is important to consider that the brain’s intrinsic network architecture that is present during the resting-state shapes the brain’s functional network architecture during task performance^53^. In other words, resting-state brain function influences task-related brain function. The observed resting-state abnormalities in our study may contribute to an impaired recruitment of these areas during functional neuroimaging tasks. For example, we found reduced resting-state rCBF in both antisocial groups in the anterior cingulate cortex (cluster two) and the pars orbitalis/orbitofrontal cortex (cluster three). Functionally, these two regions play important roles in learning from punishment cues^54,55^, changing behaviour in the face of changing contingencies^56^, and decision-making under conditions of uncertainty^57,58^ – processes which appear to be similarly impaired in those with ASPD with and without psychopathy^12^. However, such potential resting-state–functional links remain speculative in this study due to the absence of additional perfusion data obtained during functional tasks. To further disentangle which specific rCBF mechanisms underpin behavioural deficits, future studies could integrate rCBF and measurements of cerebral metabolic rate of oxygen consumption (CMRO_2_)^59^ or oxygen extraction function (OEF)^60^ during behavioural tasks to determine whether these rCBF reductions reflect metabolic deficits in ASPD subtypes beyond the resting-state.

Our precise phenotyping further enabled us to demonstrate that the ASPD+P group had significantly increased rCBF in a medial parietal cluster compared to the ASPD-P and non-offender groups. Previous studies of individuals with ASPD+P have consistently found structural^14,61^ and functional^15,17,62^ activity abnormalities in this region, especially in the posterior cingulate and precuneus. Altered grey matter volume, surface area and increased BOLD demands may underpin the observed changes in rCBF. Yet there may also be important functional explanations. Typically, this region has heightened perfusion in the resting state in healthy individuals, reflecting its role as a ‘rich club’ hub in the brain’s information processing network^21,63^. However, alterations in network topology in psychopathy^27^ could make increased function in areas like the precuneus a compensatory mechanism which underpins information integration in the disorder. This is further supported by findings that the posterior DMN does not appropriately deactivate during task engagement in people with offending history and psychopathy^64^. This region also contributes to self-/other-referential processing (including mentalizing and perspective-taking), autobiographical episodic memory, and subjective reward representation^63,65–68^. Evidence suggests that automatic mentalizing and subjective reward representational processes which draw on such areas are compromised in individuals with ASPD+P^15,69–74^. Taken together, these findings suggest that the structure and function of the posterior DMN in ASPD+P is examined in more detail in future research.

Resting-state rCBF responsivity to oxytocin also differed between the ASPD groups. Thus, OT significantly reduced rCBF in the left basal ganglia, specifically the globus pallidus and dorsal striatum (putamen and caudate), when compared to placebo, in the ASPD-P but not the ASPD+P group. Importantly, this result is unlikely to reflect non-specific vascular effects, as OT (10–40 IU) does not disrupt cerebrovascular reactivity^44^.

The basal ganglia, rich in oxytocin receptors^75^, are crucial for reinforcement learning, reward processing, habit formation, and goal-directed action selection and control^76–78^. The dorsal striatum predominantly mediates choice impulsivity, evaluating action-contingent outcomes to better select future goal-directed actions^79^. Functional MRI studies have provided evidence for dorsal striatum abnormalities in antisocial groups in childhood^80^. Youths with disruptive behaviour disorders (precursors of ASPD in adulthood) show reduced responsiveness to positive prediction errors and increased responsiveness to negative prediction errors within the dorsal striatum during feedback^81^ and reduced dorsal striatal response to early stimulus-reinforcement exposure^82^. Therefore, striatal dysfunction may underpin dysfunctional learning and decision-making in antisocial populations. Given the modulatory effect of OT on dorsal striatum function in this and other research^45,83^, and evidence for a beneficial effect of OT on reinforcement learning^84–86^, further studies investigating the potential therapeutic relevance of OT for reinforcement learning and decision-making in adults with ASPD are warranted.

We observed no significant OT effects on resting-state function in the ASPD+P group. This may be due to neurochemical differences between individuals with ASPD+P and ASPD-P. One possibility is that individuals with ASPD+P have higher peripheral endogenous OT levels^87,88^, potentially limiting the impact of exogenous administration^89^ (though see^90^. Alternatively, spatial pharmacodynamic studies show differential effects of OT on rCBF at different post-dose intervals^44,45^, and it is possible that effects in ASPD+P^17^ may have been more immediate or transient than in ASPD-P and thus not captured within our time window of 85 min post-dose. From a neurochemical perspective, such differential effects of timing between the groups might be associated with differences in oxytocin receptor genotype or availability^91,92^, or interactions with other neurotransmitters such as serotonin^93,94^. However, these conclusions are tentative and further research exploring the neurochemical underpinnings of ASPD+/-P is required.

We also did not observe significant modulatory effects of OT on resting-state brain function in the healthy non-offender group, despite previous studies demonstrating significant modulatory effects. This could be related to different time points of measurement as detailed above. Further studies utilising differing post-dose sampling time frames and different OT doses are required.

Several limitations warrant consideration. First, our study was limited to male participants, precluding conclusions about potential sex differences in OT responsivity^95^. Second, while we excluded individuals with history of traumatic brain injury (TBI) or loss of consciousness for more than 1 h, we did not exclude individuals with mild TBI. Mild TBI has been suggested to have a transient impact on perfusion^96^. Third, OT effects were measured 85 min post-dose, which is later than most fMRI studies^17,39,44,45^ including those which have demonstrated a functional impact of OT in antisocial populations. Fourth, our use of cluster-level inference and spatial smoothing meant we were only able to interpret the significance of whole clusters, and not individual voxels. While this approach is more powerful, it is also linked with a slightly lower level of spatial specificity^97^. Therefore, the findings of increased rCBF in both ASPD+/-P in the superior temporal lobe should be interpreted cautiously, and future studies may wish to use threshold-free cluster enhancement methods^98^. Finally, while we observed significant OT effects in the ASPD-P group, there was nevertheless individual variability in neural response to OT within and across the groups (Fig. 2), suggesting the need for personalized treatment approaches informed by individual level data. Larger trials incorporating genetic and endocrine markers of OT signalling may help to further enhance precision in ASPD treatment strategies.

In conclusion, we employed ASL to measure rCBF in carefully phenotyped groups of violent men with ASPD with or without psychopathy. Such men showed shared (reduced rCBF in frontotemporal regions) and distinct (increased medial parietal rCBF in the ASPD+P group in comparison to ASPD-P and non-offender groups) resting-state abnormalities. OT exerted a modulatory effect on rCBF in the left basal ganglia of the ASPD-P group. The stratification of ASPD into more biologically homogenous clinical subgroups for both mechanistic and therapeutic studies appears warranted.

## Methods

### Participants

This study included 53 male participants (31 offenders with ASPD with (*N* = 17) or without (*N* = 14) psychopathy and 22 healthy non-offenders) aged 18–60, with normal range IQ according to the Wechsler Abbreviated Scale of Intelligence (WASI-II)^99^ who consented between September 2017 and March 2020. We recruited males with convictions for violent crimes (murder, rape, attempted murder, grievous and actual bodily harm) who met DSM-5 criteria for ASPD via the National Probation Service of England and Wales and local forensic personality disorder services. We recruited healthy individuals without previous convictions from the general population through public and online advertising. All participants completed diagnostic interviews (Structured Clinical Interview for the DSM-5-Clinical and Personality Disorders (SCID-5-CV/SCID-5-PD)^1,100^ and Psychopathy Checklist-Revised (PCL-R)^3^) and authorized access to their criminal records. In line with previous research in UK samples^13–15,17,101^, we used a score of 25 as the threshold for psychopathy in this English population. We excluded participants if they had a documented or self-disclosed history – or met criteria during the diagnostic assessment – of major mental illness (bipolar 1, bipolar 2, major depression or psychotic disorders including schizophrenia, schizoaffective disorder and delusional disorder), neurological disorders, head injury resulting in loss of consciousness for 1 h or longer, severe visual or hearing impairments, or contraindications to MRI.

The study was approved by London City and East Research Ethics Committee (15/LO/1083), as well as the National Offender Management Services Research Committee (2016 − 382). After receiving a complete description of the study, all participants completed signed informed consent and were assigned anonymized subject identifiers. All assessments were conducted by an experienced research psychologist (JG) and forensic psychiatrist (JT). All research was performed in accordance with relevant guidelines and regulations and in line with the peer-reviewed and ethically approved study protocol. This trial was registered at ClinicalTrials.gov (ID NCT05383300, https://clinicaltrials.gov/study/NCT05383300) on 20/05/2022. The CONSORT checklist and flowchart can be found in the supplementary materials. Participants completed the self-report Reactive-Proactive Aggression Questionnaire. On the day of each MRI scan, participants were asked to confirm that they did not misuse substances that day and provided a urine sample to assess for substance use up to 30 days prior.

### Study design and procedure

We used a double-blind, placebo-controlled, randomised crossover design. We acquired anatomical and ASL scans as part of a larger imaging protocol conducted at the Centre for Neuroimaging Sciences, IOPPN, King’s College London (see supplementary materials for the full schedule, noting the gap between task-based scans and ASL imaging to minimise carry-over effects). For the two scanning sessions, we randomly and blindly allocated the participant to receive 40 international units (IU) of OT (Syntocinon, Novartis, Switzerland) or placebo (PL; same excipient without the oxytocin). The dose is in line with other research and has been deemed safe with no side effects^44–46,102^. Scans were scheduled at least 3 days apart to ensure complete drug washout. We counterbalanced the order of administration for OT/PL across participants, with half receiving OT first and then PL, and the other half receiving the reverse order. According to the recommendation for standardised administration^103^ and under supervision of the researcher, participants received training and then self-administered the nasal spray by snorting one puff every 30 s through alternating nostrils, for 5 min (10 puffs with 4 IU each). The ASL scans (6:23 min) were acquired on average at 84 (± 9) minutes and 85 (± 12) minutes after administration of PL and OT, respectively. This time delay is referred to as the variable ‘minutes since dose’. Scans were acquired at the same time of the day within and between participants (see supplementary materials for details).

### Image acquisition

We used a General Electric MR750 3Tesla MRI scanner and 32-channel C-RMNova head coil for this study. During each session, we acquired a 3-dimensional pseudo-continuous ASL scan (60 slice partitions with thickness and gap = 3 mm, TE = 1109 ms, TR = 5180 ms, flip angle = 111°, FOV = 240 × 240 mm^2^, in-plane resolution = 3.6 mm; radiofrequency inversion pulse = 1825 ms, delay = 2025 ms, control-label image pairs = 5). The acquisition of the final rCBF map for each participant at each session was in line with recommendations^104^.

We also acquired a 3D high-resolution T1-weighted whole-brain anatomical image during each scanning session (MPRAGE, 196 slices with thickness and gap = 1.2 mm, TE = 3.02 ms, TR = 7.31 ms, TI = 400 ms, FA = 11°, FOV = 270 × 270 mm^2^, matrix = 256 × 256, voxel resolution = 1.05 × 1.05 × 1.2 mm^3^).

### Image pre-processing

We confirmed good image quality and presence of typical perfusion values ranging between 20 and 110 ml/100 g/minute across the whole brain, indicating accurate computation of CBF maps^104^, using FSLeyes. We pre-processed the scans in the Automatic Software for ASL Processing (ASAP) toolbox, version 4.0 in Matlab 2018b. Steps included (1) co-registration of proton density and T1 images; (2) subject-specific normalization of CBF maps; (3) skull-stripping, segmentation and removal of extra-cerebral signal from the normalized CBF maps; (4) partial volume correction and normalization of the CBF map to MNI152 space; and (5) 8 mm Gaussian spatial smoothing of the CBF map. An explicit grey matter tissue probability mask (20%), derived by thresholding the FSL grey matter template at 0.20, was applied. We calculated global median CBF for each participant and each session and included this in statistical analysis as a covariate of no-interest to improve signal-to-noise ratio and the sensitivity of within-subject changes in local areas. We used median rCBF values throughout due to a skew towards the lower end of the typical range.

### Statistical analysis

We compared demographic and clinical characteristics, as well as global median CBF using ANOVA, ANCOVA, and Chi-squared tests, or non-parametric equivalents when normality assumptions were not met, in SPSSv29. We interpreted significant main effects via Sidak-corrected pairwise comparisons.

For the a priori ROI analysis, we extracted median rCBF from the bilateral amygdalae and anterior insulae using Harvard-Oxford atlas masks in MNI space. We assessed main effects of group, treatment, and their interaction with bootstrapped linear mixed models in JASP, covarying for global CBF, age, and minutes since dose. We applied FDR correction for multiple comparisons across the ROIs. To further evaluate the robustness of our findings, we conducted Bayesian linear mixed models post hoc (see supplementary methods).

We conducted whole-brain analyses in SPM12 (www.fil.ion.ucl.ac.uk/spm) using a partitioned errors approach to accommodate repeated-measures design assumptions^105^. We examined the main effect of group using a one-way ANOVA on individual-averaged OT and PL CBF maps. We assessed the main effect of treatment via a one-sample t-test using difference images, subtracting an individual’s PL CBF map from their OT CBF map. We tested the group by treatment interaction effect with a one-way ANOVA on the difference images. We covaried for global CBF, age, and minutes since dose in all models. We applied F-contrasts to assess main and interaction effects, with cluster-level inference using a cluster-forming threshold of *p* = 0.005 and family-wise error correction at α = 0.05; in accordance with prior ASL studies investigating OT effects^44,45,106^. For significant clusters, we extracted and compared raw median CBF values using post-hoc pairwise comparisons or simple main effects tests with the Sidak correction for multiple comparisons in SPSSv29.

## Supplementary Information

Below is the link to the electronic supplementary material.

Supplementary Material 1

## Data Availability

The datasets generated during and/or analysed during the current study are available from the corresponding author on reasonable request.

## References

[1] First, M. B., Williams, J. B. W., Benjamin, L. S. & Spitzer, R. L. *User’s Guide for the SCID-5-PD (Structured Clinical Interview for DSM-5 Personality Disorder)* (American Psychiatric Association, 2015).

[2] De Brito, S. A. et al. Psychopathy. *Nat. Rev. Dis. Primers*. **7**, 1–21 (2021).33414454

[3] Hare, R. D. *Manual for the Hare Psychopathy Checklist-Revised* (Guilford, 1991). 10.1007/978-0-387-79948-3_837

[4] Moffitt, T. E. Male antisocial behaviour in adolescence and beyond. *Nat. Hum. Behav.* **2**, 177–186 (2018). 30271880 PMC6157602

[5] Azevedo, J., Vieira-Coelho, M., Castelo-Branco, M., Coelho, R. & Figueiredo-Braga, M. Impulsive and premeditated aggression in male offenders with antisocial personality disorder. *PLoS One*. **15**, e0229876 (2020). 32142531 10.1371/journal.pone.0229876 PMC7059920

[6] Riser, R. E. & Kosson, D. S. Criminal behavior and cognitive processing in male offenders with antisocial personality disorder with and without comorbid psychopathy. *Personality Disorders: Theory Res. Treat.* **4**, 332–340 (2013). 10.1037/a0033303 24378159

[7] Olver, M. E., Lewis, K. & Wong, S. C. P. Risk reduction treatment of High-Risk psychopathic offenders: the relationship of psychopathy and treatment change to violent recidivism. *Personality Disorders: Theory Res. Treat.* **4**, 160–167 (2013). 10.1037/a0029769 23046041

[8] Kosson, D. S., Lorenz, A. R. & Newman, J. P. Effects of comorbid psychopathy on criminal offending and emotion processing in male offenders with antisocial personality disorder. *J. Abnorm. Psychol.* **115**, 798–806 (2006). 17100537 10.1037/0021-843X.115.4.798

[9] Mayer, S. V., Jusyte, A., Klimecki-Lenz, O. M. & Schönenberg, M. Empathy and altruistic behavior in antisocial violent offenders with psychopathic traits. *Psychiatry Res.* **269**, 625–632 (2018). 30208352 10.1016/j.psychres.2018.08.035

[10] Shepherd, S. M., Campbell, R. E., Ogloff, J. R. P. & Psychopathy Antisocial personality Disorder, and reconviction in an Australian sample of forensic patients. *Int. J. Offender Ther. Comp. Criminol.* **62**, 609–628 (2018). 27288398 10.1177/0306624X16653193

[11] Dugré, J. R. et al. Neurofunctional abnormalities in antisocial spectrum: A meta-analysis of fMRI studies on five distinct neurocognitive research domains. *Neurosci. Biobehav Rev.* **119**, 168–183 (2020). 32956690 10.1016/j.neubiorev.2020.09.013

[12] De Brito, S. A., Viding, E., Kumari, V., Blackwood, N. & Hodgins, S. Cool and hot executive function impairments in violent offenders with antisocial personality disorder with and without psychopathy. *PLoS One*. **8**, e65566 (2013). 23840340 10.1371/journal.pone.0065566 PMC3688734

[13] Tully, J. et al. Impaired striatal glutamate/GABA regulation in violent offenders with antisocial personality disorder and psychopathy. *Mol. Psychiatry*. **2024**, 1–9. 10.1038/s41380-024-02437-4 (2024). 10.1038/s41380-024-02437-4 PMC11371654 38326560

[14] Gregory, S. et al. The antisocial brain: psychopathy matters. A structural MRI investigation of antisocial male violent offenders. *Arch. Gen. Psychiatry*. **69**, 962–972 (2012). 22566562 10.1001/archgenpsychiatry.2012.222

[15] Gregory, S. et al. Punishment and psychopathy: a case-control functional MRI investigation of reinforcement learning in violent antisocial personality disordered men. *Lancet Psychiatry*. **2**, 153–160 (2015). 26359751 10.1016/S2215-0366(14)00071-6

[16] Decety, J., Skelly, L. R. & Kiehl, K. A. Brain response to empathy-eliciting scenarios involving pain in incarcerated individuals with psychopathy. *JAMA Psychiatry*. **70**, 638–645 (2013). 23615636 10.1001/jamapsychiatry.2013.27 PMC3914759

[17] Tully, J. et al. Oxytocin normalises the implicit processing of fearful faces in psychopathy: a randomised crossover study using fMRI. *Nat. Mental Health*. **1**, 420–427 (2023). 38665476 10.1038/s44220-023-00067-3 PMC11041724

[18] Finn, E. S. Is it time to put rest to rest? *Trends Cogn. Sci.* **25**, 1021–1032 (2021). 34625348 10.1016/j.tics.2021.09.005 PMC8585722

[19] Fox, M. D. & Greicius, M. Clinical applications of resting state functional connectivity. *Front. Syst. Neurosci.* **4**, 1–13 (2010). 20592951 10.3389/fnsys.2010.00019 PMC2893721

[20] Menon, V. Large-scale brain networks and psychopathology: a unifying triple network model. *Trends Cogn. Sci.* **15**, 483–506 (2011). 21908230 10.1016/j.tics.2011.08.003

[21] Gusnard, D. A. & Raichle, M. E. Searching for a baseline: functional imaging and the resting human brain. *Nat. Rev. Neurosci.* **2**, 685–694 (2001). 11584306 10.1038/35094500

[22] Moutoussis, M. et al. Decision-making ability, psychopathology, and brain connectivity. *Neuron* **109**, 2025–2040 (2021). 34019810 10.1016/j.neuron.2021.04.019 PMC8221811

[23] Tavor, I. et al. Task-free MRI predicts individual differences in brain activity during task performance. *Sci. (1979)*. **352**, 216–220 (2016). 10.1126/science.aad8127 PMC6309730 27124457

[24] Molloy, M. F. et al. Regional, but not brain-wide, graph theoretic measures are robustly and reproducibly linked to general cognitive ability. *Cereb. Cortex* **35** (2025). 10.1093/cercor/bhaf074 PMC13017514 40211548

[25] Espinoza, F. A. et al. Aberrant functional network connectivity in psychopathy from a large (N = 985) forensic sample. *Hum. Brain Mapp.* **39**, 2624–2634 (2018). 29498761 10.1002/hbm.24028 PMC5951759

[26] Contreras-Rodríguez, O. et al. Functional connectivity bias in the prefrontal cortex of psychopaths. *Biol. Psychiatry*. **78**, 647–655 (2015). 24742618 10.1016/j.biopsych.2014.03.007

[27] Tillem, S. et al. Psychopathy is associated with shifts in the organization of neural networks in a large incarcerated male sample. *Neuroimage Clin.* **24**, 1–12 (2019). 10.1016/j.nicl.2019.102083 PMC6861623 31795050

[28] Jiang, W. et al. Disrupted functional connectome in antisocial personality disorder. *Brain Imaging Behav.* **11**, 1071–1084 (2017). 27541949 10.1007/s11682-016-9572-z PMC5362344

[29] Tang, Y., Jiang, W., Liao, J., Wang, W. & Luo, A. Identifying individuals with antisocial personality disorder using Resting-State fMRI. *PLoS One*. **8**, e60652 (2013). 23593272 10.1371/journal.pone.0060652 PMC3625191

[30] Simon, A. B. & Buxton, R. B. Understanding the dynamic relationship between cerebral blood flow and the BOLD signal: implications for quantitative functional MRI. *Neuroimage* **116**, 158–167 (2015). 25862267 10.1016/j.neuroimage.2015.03.080 PMC4468003

[31] Stewart, S. B., Koller, J. M., Campbell, M. C. & Black, K. J. Arterial spin labeling versus BOLD in direct challenge and drug-task interaction Pharmacological fMRI. *PeerJ* **2**, 1–14 (2014). 10.7717/peerj.687 PMC4266850 25538867

[32] Kolla, N. J. & Houle, S. Single-Photon emission computed tomography and positron emission tomography studies of antisocial personality disorder and aggression: a targeted review. *Curr. Psychiatry Rep.* **21**, 1–11 (2019). 30852703 10.1007/s11920-019-1011-6 PMC6440931

[33] Soderstrom, H., Tullberg, M., Wikkelsö, C., Ekholm, S. & Forsman, A. Reduced regional cerebral blood flow in non-psychotic violent offenders. *Psychiatry Res. Neuroimaging*. **98**, 29–41 (2000). 10.1016/s0925-4927(99)00049-9 10708924

[34] Alia-Klein, N. et al. Reactions to media violence: it’s in the brain of the beholder. *PLoS One*. **9**, e107260 (2014). 25208327 10.1371/journal.pone.0107260 PMC4160225

[35] Borogovac, A. & Asllani, I. Arterial spin labeling (ASL) fMRI: Advantages, theoretical constrains and experimental challenges in neuroscience. *Int J Biomed Imaging* **2012**, 1–13 (2012). 10.1155/2012/818456 PMC3432878 22966219

[36] Hodkinson, D. J. et al. Quantifying the test–retest reliability of cerebral blood flow measurements in a clinical model of on-going post-surgical pain: A study using pseudo-continuous arterial spin labelling. *Neuroimage Clin.* **3**, 301–310 (2013). 24143296 10.1016/j.nicl.2013.09.004 PMC3797555

[37] Menon, R. & Neumann, I. D. Detection, processing and reinforcement of social cues: regulation by the Oxytocin system. *Nat. Rev. Neurosci.* **24**, 761–777 (2023). 37891399 10.1038/s41583-023-00759-w

[38] Yao, S. & Kendrick, K. M. How does oxytocin modulate human behavior?. *Mol. Psychiatry*10.1038/s41380-025-02898-1 (2025). 39827220 10.1038/s41380-025-02898-1

[39] Jeung-Maarse, H., Schmitgen, M. M., Schmitt, R., Bertsch, K. & Herpertz, S. C. Oxytocin effects on amygdala reactivity to angry faces in males and females with antisocial personality disorder. *Neuropsychopharmacology* **48**, 946–953 (2023). 36941365 10.1038/s41386-023-01549-9 PMC10156793

[40] Whelan, T. P. et al. Editorial perspective: bridging the translational neuroscience gap in autism - development of the ‘shiftability’ paradigm. *J. Child. Psychol. Psychiatry*. **65**, 862–865 (2024). 38130022 10.1111/jcpp.13940

[41] Bloomfield, M. A. P. et al. The effects of acute Cannabidiol on cerebral blood flow and its relationship to memory: an arterial spin labelling magnetic resonance imaging study. *J. Psychopharmacol.* **34**, 981–989 (2020). 32762272 10.1177/0269881120936419 PMC7436497

[42] Bryant, J. E. et al. Ketamine induced changes in regional cerebral blood flow, interregional connectivity patterns, and glutamate metabolism. *J. Psychiatr Res.* **117**, 108–115 (2019). 31376621 10.1016/j.jpsychires.2019.07.008 PMC7291620

[43] Martens, M. A. G. et al. Dopaminergic modulation of regional cerebral blood flow: an arterial spin labelling study of genetic and Pharmacological manipulation of COMT activity. *Neuroimage* **234**, 117999 (2021). 33789133 10.1016/j.neuroimage.2021.117999

[44] Martins, D. et al. Effects of route of administration on oxytocin-induced changes in regional cerebral blood flow in humans. *Nat. Commun.* **11**, 1–16 (2020). 32127545 10.1038/s41467-020-14845-5 PMC7054359

[45] Paloyelis, Y. et al. A Spatiotemporal profile of in vivo cerebral blood flow changes following intranasal Oxytocin in humans. *Biol. Psychiatry*. **79**, 693–705 (2016). 25499958 10.1016/j.biopsych.2014.10.005

[46] Martins, D. et al. Less is more’: a dose-response account of intranasal Oxytocin pharmacodynamics in the human brain. *Prog Neurobiol.* **211**, 1–17 (2022). 10.1016/j.pneurobio.2022.102239 35122880

[47] Tully, J. et al. A systematic review and meta-analysis of brain volume abnormalities in disruptive behaviour disorders, antisocial personality disorder and psychopathy. *Nat. Mental Health*. **1**, 163–173 (2023).

[48] De Brito, S. A., McDonald, D., Camilleri, J. A. & Rogers, J. C. Cortical and subcortical Gray matter volume in psychopathy: a voxel-wise meta-analysis. *J. Abnorm. Psychol.* **130**, 627–640 (2021). 34553958 10.1037/abn0000698

[49] Coid, J. et al. The co-morbidity of personality disorder and clinical syndromes in prisoners. *Criminal Behav. Mental Health*. **19**, 321–333 (2009). 10.1002/cbm.747 19908330

[50] Trull, T. J., Jahng, S., Tomko, R. L., Wood, P. K. & Sher, K. J. Revised NESARC personality disorder diagnoses: Gender, prevalence, and comorbidity with substance dependence disorders. *J. Pers. Disord*. **24**, 412–426 (2010). 20695803 10.1521/pedi.2010.24.4.412 PMC3771514

[51] Deming, P., Heilicher, M. & Koenigs, M. How reliable are amygdala findings in psychopathy? A systematic review of MRI studies. *Neurosci. Biobehav Rev.* **142**, 1–18 (2022). 10.1016/j.neubiorev.2022.104875 36116578

[52] Murphy, K. et al. Pulsed arterial spin labeling perfusion imaging at 3 T: estimating the number of subjects required in common designs of clinical trials. *Magn. Reson. Imaging*. **29**, 1382–1389 (2011). 21546190 10.1016/j.mri.2011.02.030

[53] Cole, M. W., Bassett, D. S., Power, J. D., Braver, T. S. & Petersen, S. E. Intrinsic and task-evoked network architectures of the human brain. *Neuron* **83**, 238–251 (2014). 24991964 10.1016/j.neuron.2014.05.014 PMC4082806

[54] Xue, G. et al. Common neural mechanisms underlying reversal learning by reward and punishment. *PLoS One*. **8**, e82169 (2013). 24349211 10.1371/journal.pone.0082169 PMC3859585

[55] Elster, E. M. et al. Altered neural responses to punishment learning in conduct disorder. *Biol. Psychiatry Cogn. Neurosci. Neuroimaging*. 10.1016/J.BPSC.2025.01.003 (2025). 39805552 10.1016/j.bpsc.2025.01.003

[56] Uddin, L. Q. Cognitive and behavioural flexibility: neural mechanisms and clinical considerations. *Nat. Rev. Neurosci.* **22**, 167–179 (2021). 33536614 10.1038/s41583-021-00428-w PMC7856857

[57] Hsu, M., Bhatt, M., Adolphs, R., Tranel, D. & Camerer, C. F. Neural systems responding to degrees of uncertainty in human decision-making. *Sci. (1979)*. **310**, 1680–1683 (2005). 10.1126/science.1115327 16339445

[58] Klein-Flügge, M. C., Bongioanni, A. & Rushworth, M. F. S. Medial and orbital frontal cortex in decision-making and flexible behavior. *Neuron* **110**, 2743–2770 (2022). 35705077 10.1016/j.neuron.2022.05.022 PMC7618973

[59] Germuska, M. & Wise, R. G. Calibrated fMRI for mapping absolute CMRO2: practicalities and prospects. *Neuroimage* **187**, 145–153 (2019). 29605580 10.1016/j.neuroimage.2018.03.068

[60] Singh, N. et al. The effects of acute methylene blue administration on cerebral blood flow and metabolism in humans and rats. *J. Cereb. Blood Flow Metab.* **43**, 95–105 (2023). 36803299 10.1177/0271678X231157958 PMC10638993

[61] Sethi, A. et al. Emotional detachment in psychopathy: involvement of dorsal default-mode connections. *Cortex* **62**, 11–19 (2015). 25218645 10.1016/j.cortex.2014.07.018

[62] Deming, P. & Koenigs, M. Functional neural correlates of psychopathy: a meta-analysis of MRI data. *Transl Psychiatry*. **10**, 1–8 (2020). 32376864 10.1038/s41398-020-0816-8 PMC7203015

[63] Dadario, N. B. & Sughrue, M. E. The functional role of the precuneus. *Brain* **146**, 3598–3607 (2023). 37254740 10.1093/brain/awad181

[64] Freeman, S. M. et al. The posteromedial region of the default mode network shows attenuated task-induced deactivation in psychopathic prisoners. *Neuropsychology* **29**, 493–500 (2015). 25133317 10.1037/neu0000118 PMC4333113

[65] Cavanna, A. E. & Trimble, M. R. The precuneus: a review of its functional anatomy and behavioural correlates. *Brain* **129**, 564–583 (2006). 16399806 10.1093/brain/awl004

[66] Kable, J. W. & Glimcher, P. W. The neural correlates of subjective value during intertemporal choice. *Nat. Neurosci.* **10**, 1625–1633 (2007). 17982449 10.1038/nn2007 PMC2845395

[67] Liu, X., Hairston, J., Schrier, M. & Fan, J. Common and distinct networks underlying reward Valence and processing stages: A meta-analysis of functional neuroimaging studies. *Neurosci. Biobehav Rev.* **35**, 1219–1236 (2011). 21185861 10.1016/j.neubiorev.2010.12.012 PMC3395003

[68] Arioli, M., Cattaneo, Z., Ricciardi, E. & Canessa, N. Overlapping and specific neural correlates for empathizing, affective mentalizing, and cognitive mentalizing: A coordinate-based meta-analytic study. *Hum. Brain Mapp.* **42**, 4777–4804 (2021). 34322943 10.1002/hbm.25570 PMC8410528

[69] Drayton, L. A., Santos, L. R. & Baskin-Sommers, A. R. Psychopaths fail to automatically take the perspective of others. *Proc. Natl. Acad. Sci. U S A*. **115**, 3302–3307 (2018). 29531085 10.1073/pnas.1721903115 PMC5879707

[70] Deming, P. et al. Psychopathic traits linked to alterations in neural activity during personality judgments of self and others. *Neuroimage Clin.* **18**, 575–581 (2018). 29845005 10.1016/j.nicl.2018.02.029 PMC5964831

[71] Deming, P. et al. Psychopathy is associated with fear-specific reductions in neural activity during affective perspective-taking. *Neuroimage* **223**, 117342 (2020). 32898678 10.1016/j.neuroimage.2020.117342 PMC9831240

[72] Newbury-Helps, J., Feigenbaum, J. & Fonagy, P. Offenders with antisocial personality disorder display more impairments in mentalizing. *J. Pers. Disord*. **31**, 232–255 (2017). 27064853 10.1521/pedi_2016_30_246

[73] Taubner, S., White, L. O., Zimmermann, J., Fonagy, P. & Nolte, T. Attachment-related mentalization moderates the relationship between psychopathic traits and proactive aggression in adolescence. *J. Abnorm. Child. Psychol.* **41**, 929–938 (2013). 23512713 10.1007/s10802-013-9736-x

[74] Bigot, A. et al. Confusing my viewpoint with his: altered self-other distinction performance in antisocial personality disorder. *Personal Disord*. **16**, 110–121 (2025). 39760725 10.1037/per0000660

[75] Quintana, D. S. et al. Oxytocin pathway gene networks in the human brain. *Nat. Commun.* **10**, 1–12 (2019). 30737392 10.1038/s41467-019-08503-8 PMC6368605

[76] Balleine, B. W., Delgado, M. R. & Hikosaka, O. The role of the dorsal striatum in reward and Decision-Making. *J. Neurosci.* **27**, 8161 (2007). 17670959 10.1523/JNEUROSCI.1554-07.2007 PMC6673072

[77] Malvaez, M. & Wassum, K. M. Regulation of habit formation in the dorsal striatum. *Curr. Opin. Behav. Sci.* **20**, 67–74 (2018). 29713658 10.1016/j.cobeha.2017.11.005 PMC5920535

[78] Haber, S. N. & Behrens, T. E. J. The neural network underlying Incentive-Based learning: implications for interpreting circuit disruptions in psychiatric disorders. *Neuron* **83**, 1019–1039 (2014). 25189208 10.1016/j.neuron.2014.08.031 PMC4255982

[79] Kim, B. S. & Im, H. I. The role of the dorsal striatum in choice impulsivity. *Ann. N Y Acad. Sci.* **1451**, 92–111 (2019). 30277562 10.1111/nyas.13961

[80] Hawes, S. W. et al. Reward processing in children with disruptive behavior disorders and Callous-Unemotional traits in the ABCD study. *Am. J. Psychiatry*. **178**, 333–342 (2021). 32731811 10.1176/appi.ajp.2020.19101092 PMC7855017

[81] White, S. F. et al. Disrupted expected value and prediction error signaling in youths with disruptive behavior disorders during a passive avoidance task. *Am. J. Psychiatry*. **170**, 315–323 (2013). 23450288 10.1176/appi.ajp.2012.12060840 PMC3941772

[82] Finger, E. C. et al. Disrupted reinforcement signaling in the orbitofrontal cortex and caudate in youths with conduct disorder or oppositional defiant disorder and a high level of psychopathic traits. *Am. J. Psychiatry*. **168**, 152–162 (2011). 21078707 10.1176/appi.ajp.2010.10010129 PMC3908480

[83] Zhao, Z. et al. Oxytocin differentially modulates specific dorsal and ventral striatal functional connections with frontal and cerebellar regions. *Neuroimage* **184**, 781–789 (2019). 30266264 10.1016/j.neuroimage.2018.09.067

[84] Zhuang, Q. et al. Oxytocin-induced facilitation of learning in a probabilistic task is associated with reduced feedback- and error-related negativity potentials. *J. Psychopharmacol.* **35**, 40–49 (2021). 33274683 10.1177/0269881120972347

[85] Kruppa, J. A. et al. Neural modulation of social reinforcement learning by intranasal Oxytocin in male adults with high-functioning autism spectrum disorder: a randomized trial. *Neuropsychopharmacology* **44**, 749–756 (2019). 30390065 10.1038/s41386-018-0258-7 PMC6372686

[86] Martins, D., Lockwood, P., Cutler, J., Moran, R. & Paloyelis, Y. Oxytocin modulates neurocomputational mechanisms underlying prosocial reinforcement learning. *Prog Neurobiol.* **213**, 1–15 (2022). 10.1016/j.pneurobio.2022.102253 35248585

[87] Berends, Y. R. et al. Oxytocin and vasopressin in male forensic psychiatric patients with personality disorders and healthy controls. *J. Forens Psychiatry Psychol.* **33**, 130–151 (2022).

[88] Mitchell, I. J. et al. Psychopathic characteristics are related to high basal urinary Oxytocin levels in male forensic patients. *J. Forensic Psychiatry Psychol.* **24**, 309–318 (2013).

[89] Carson, D. S. et al. Cerebrospinal fluid and plasma Oxytocin concentrations are positively correlated and negatively predict anxiety in children. *Mol. Psychiatry*. **20**, 1085–1090 (2014). 25349162 10.1038/mp.2014.132

[90] Martins, D., Gabay, A. S., Mehta, M. & Paloyelis, Y. Salivary and plasmatic Oxytocin are not reliable trait markers of the physiology of the Oxytocin system in humans. *Elife* **9**, 1–19 (2020). 10.7554/eLife.62456 PMC7732341 33306025

[91] Seeley, S. H., Chou, Y. & O’Connor, M. F. Intranasal Oxytocin and OXTR genotype effects on resting state functional connectivity: A systematic review. *Neurosci. Biobehav Rev.* **95**, 17–32 (2018). 30243577 10.1016/j.neubiorev.2018.09.011

[92] Kou, J. et al. A randomized trial shows dose-frequency and genotype May determine the therapeutic efficacy of intranasal Oxytocin. *Psychol. Med.* **52**, 1959–1968 (2022). 33272333 10.1017/S0033291720003803

[93] Mottolese, R., Redout́e, J., Costes, N., Le Bars, D. & Sirigu, A. Switching brain serotonin with Oxytocin. *Proc. Natl. Acad. Sci. U S A*. **111**, 8637–8642 (2014). 24912179 10.1073/pnas.1319810111 PMC4060712

[94] Wang, J. et al. Effects of exogenous oxytocin on human brain function are regulated by oxytocin gene expression: a meta-analysis of 20 years of oxytocin neuroimaging and transcriptomic analyses. *Neurosci. Biobehav Rev.*10.1016/J.NEUBIOREV.2025.106478 (2025). 41260504 10.1016/j.neubiorev.2025.106478

[95] Dumais, K. M., Kulkarni, P. P., Ferris, C. F. & Veenema, A. H. Sex differences in neural activation following different routes of Oxytocin administration in awake adult rats. *Psychoneuroendocrinology* **81**, 52–62 (2017). 28412582 10.1016/j.psyneuen.2017.04.003 PMC5497485

[96] Stephens, J. A., Liu, P., Lu, H. & Suskauer, S. J. Cerebral blood flow after mild traumatic brain injury: associations between symptoms and Post-Injury perfusion. *J. Neurotrauma*. **35**, 241 (2018). 28967326 10.1089/neu.2017.5237 PMC5784789

[97] Eklund, A., Nichols, T. E. & Knutsson, H. Cluster failure: why fMRI inferences for Spatial extent have inflated false-positive rates. *Proc. Natl. Acad. Sci. U S A*. **113**, 7900–7905 (2016). 27357684 10.1073/pnas.1602413113 PMC4948312

[98] Smith, S. M. & Nichols, T. E. Threshold-free cluster enhancement: addressing problems of smoothing, threshold dependence and localisation in cluster inference. *Neuroimage* **44**, 83–98 (2009). 18501637 10.1016/j.neuroimage.2008.03.061

[99] Wechsler, D. *Wechlser Abbreviated Scale of Intelligence, Second Edition (WASI-II)* (NCS Pearson, 2011). 10.1177/0734282912467756

[100] First, M. B., Williams, J. B. W., Karg, R. S. & Spitzer, R. L. *User’s Guide To Structured Clinical Interview for DSM-5 Disorders, Clinical Version* (American Psychiatric Association, 2016).

[101] Cooke, D. J. & Michie, C. Psychopathy across cultures: North America and Scotland compared. *J. Abnorm. Psychol.* **108**, 58–68 (1999). 10066993 10.1037//0021-843x.108.1.58

[102] MacDonald, E. et al. A review of safety, side-effects and subjective reactions to intranasal Oxytocin in human research. *Psychoneuroendocrinology* **36**, 1114–1126 (2011). 21429671 10.1016/j.psyneuen.2011.02.015

[103] Guastella, A. J. et al. Recommendations for the standardisation of Oxytocin nasal administration and guidelines for its reporting in human research. *Psychoneuroendocrinology* **38**, 612–625 (2013). 23265311 10.1016/j.psyneuen.2012.11.019

[104] Alsop, D. C. et al. Recommended implementation of arterial spin-labeled perfusion MRI for clinical applications: A consensus of the ISMRM perfusion study group and the European consortium for ASL in dementia. *Magn. Reson. Med.* **73**, 102–116 (2015). 24715426 10.1002/mrm.25197 PMC4190138

[105] McFarquhar, M. et al. Multivariate and repeated measures (MRM): A new toolbox for dependent and multimodal group-level neuroimaging data. *Neuroimage* **132**, 373–389 (2016). 26921716 10.1016/j.neuroimage.2016.02.053 PMC4862963

[106] Martins, D. et al. Investigating resting brain perfusion abnormalities and disease target-engagement by intranasal Oxytocin in women with bulimia nervosa and binge-eating disorder and healthy controls. *Transl Psychiatry*. **10**, 1–13 (2020). 32513936 10.1038/s41398-020-00871-w PMC7280271

