# Supplementary material for: Resting-state brain function and its modulation by intranasal oxytocin in antisocial personality disorder with and without psychopathy
Source: Sci Rep. 2026 Jan 25;16:6207. doi: 10.1038/s41598-026-36661-5 (PMC12905299; doi:10.1038/s41598-026-36661-5)
Supplement: Supplementary file 1 — Supplementary Material 1 [file 41598_2026_36661_MOESM1_ESM.pdf]

Supplementary methods

CONSORT checklist and flowchart

CONSORT Checklist

Section/topic	No	CONSORT 2025 checklist item description	Reported on page no.
**Title and abstract**			
Title and structured abstract	1a	Identification as a randomised trial	11
	1b	Structured summary of the trial design, methods, results, and conclusions	2
**Open science**			
Trial registration	2	Name of trial registry, identifying number (with URL) and date of registration	11
Protocol and statistical analysis plan	3	Where the trial protocol and statistical analysis plan can be accessed	11
Data sharing	4	Where and how the individual de-identified participant data (including data dictionary), statistical code and any other materials can be accessed	20
Funding and conflicts of interest	5a	Sources of funding and other support (eg, supply of drugs), and role of funders in the design, conduct, analysis and reporting of the trial	20
	5b	Financial and other conflicts of interest of the manuscript authors	20
**Introduction**			
Background and rationale	6	Scientific background and rationale	3-5
Objectives	7	Specific objectives related to benefits and harms	4-5
**Methods**			
Patient and public involvement	8	Details of patient or public involvement in the design, conduct and reporting of the trial	N/A
Trial design	9	Description of trial design including type of trial (eg, parallel group, crossover), allocation ratio, and framework (eg, superiority, equivalence, non-inferiority, exploratory)	11
Changes to trial protocol	10	Important changes to the trial after it commenced including any outcomes or analyses that were not prespecified, with reason	N/A
Trial setting	11	Settings (eg, community, hospital) and locations (eg, countries, sites) where the trial was conducted	11
Eligibility criteria	12a	Eligibility criteria for participants	10
	12b	If applicable, eligibility criteria for sites and for individuals delivering the interventions (eg, surgeons, physiotherapists)	N/A
Intervention and comparator	13	Intervention and comparator with sufficient details to allow replication. If relevant, where additional materials describing the intervention and comparator (eg, intervention manual) can be accessed	10-11

Outcomes	14	Prespecified primary and secondary outcomes, including the specific measurement variable (eg, systolic blood pressure), analysis metric (eg, change from baseline, final value, time to event), method of aggregation (eg, median, proportion), and time point for each outcome	10-13
Harms	15	How harms were defined and assessed (eg, systematically, non-systematically)	N/A
Sample size	16a	How sample size was determined, including all assumptions supporting the sample size calculation	Suppl Mat, 5
	16b	Explanation of any interim analyses and stopping guidelines	N/A
Randomisation:			
Sequence generation	17a	Who generated the random allocation sequence and the method used	Suppl Mat, 5
	17b	Type of randomisation and details of any restriction (eg, stratification, blocking and block size)	Suppl Mat, 5
			**Reported on page no.**
Allocation concealment mechanism	18	Mechanism used to implement the random allocation sequence (eg, central computer/telephone; sequentially numbered, opaque, sealed containers), describing any steps to conceal the sequence until interventions were assigned	Suppl Mat, 5
Implementation	19	Whether the personnel who enrolled and those who assigned participants to the interventions had access to the random allocation sequence	Suppl Mat, 5
Blinding	20a	Who was blinded after assignment to interventions (eg, participants, care providers, outcome assessors, data analysts)	11 & Suppl Mat 5
	20b	If blinded, how blinding was achieved and description of the similarity of interventions	Suppl Mat, 5
Statistical methods	21a	Statistical methods used to compare groups for primary and secondary outcomes, including harms	12-13
	21b	Definition of who is included in each analysis (eg, all randomised participants), and in which group	10
	21c	How missing data were handled in the analysis	21
	21d	Methods for any additional analyses (eg, subgroup and sensitivity analyses), distinguishing prespecified from post hoc	12-13 & Suppl Mat 5-6 and 7-8
**Results**			
Participant flow, including flow diagram	22a	For each group, the numbers of participants who were randomly assigned, received intended intervention, and were analysed for the primary outcome	10
	22b	For each group, losses and exclusions after randomisation, together with reasons	Flowchart, Suppl Mat 4
Recruitment	23a	Dates defining the periods of recruitment and follow-up for outcomes of benefits and harms	10
	23b	If relevant, why the trial ended or was stopped	N/A
Intervention and comparator delivery	24a	Intervention and comparator as they were actually administered (eg, where appropriate, who delivered the intervention/comparator, how participants adhered, whether they were delivered as intended (fidelity))	11
	24b	Concomitant care received during the trial for each group	N/A

Baseline data	25	A table showing baseline demographic and clinical characteristics for each group	Table 1
Numbers analysed, outcomes and estimation	26	For each primary and secondary outcome, by group:<br>● the number of participants included in the analysis<br>● the number of participants with available data at the outcome time point<br>● result for each group, and the estimated effect size and its precision (such as 95% confidence interval)<br>● for binary outcomes, presentation of both absolute and relative effect size	Table 2 & p. 6
Harms	27	All harms or unintended events in each group	N/A
Ancillary analyses	28	Any other analyses performed, including subgroup and sensitivity analyses, distinguishing pre-specified from post hoc	6 & Suppl Mat 6-8
**Discussion**			
Interpretation	29	Interpretation consistent with results, balancing benefits and harms, and considering other relevant evidence	6-10
Limitations	30	Trial limitations, addressing sources of potential bias, imprecision, generalisability, and, if relevant, multiplicity of analyses	9-10

Citation: Hopewell S, Chan AW, Collins GS, Hróbjartsson A, Moher D, Schulz KF, et al. CONSORT 2025 Statement: updated guideline for reporting randomised trials. BMJ. 2025; 388:e081123. https://dx.doi.org/10.1136/bmj-2024-081123
© 2025 Hopewell et al. This is an Open Access article distributed under the terms of the Creative Commons Attribution License (https://creativecommons.org/licenses/by/4.0/), which permits unrestricted use, distribution, and reproduction in any medium, provided the original work is properly cited.

*We strongly recommend reading this statement in conjunction with the CONSORT 2025 Explanation and Elaboration and/or the CONSORT 2025 Expanded Checklist for important clarifications on all the items. We also recommend reading relevant CONSORT extensions. See www.consort-spirit.org.

CONSORT Flowchart

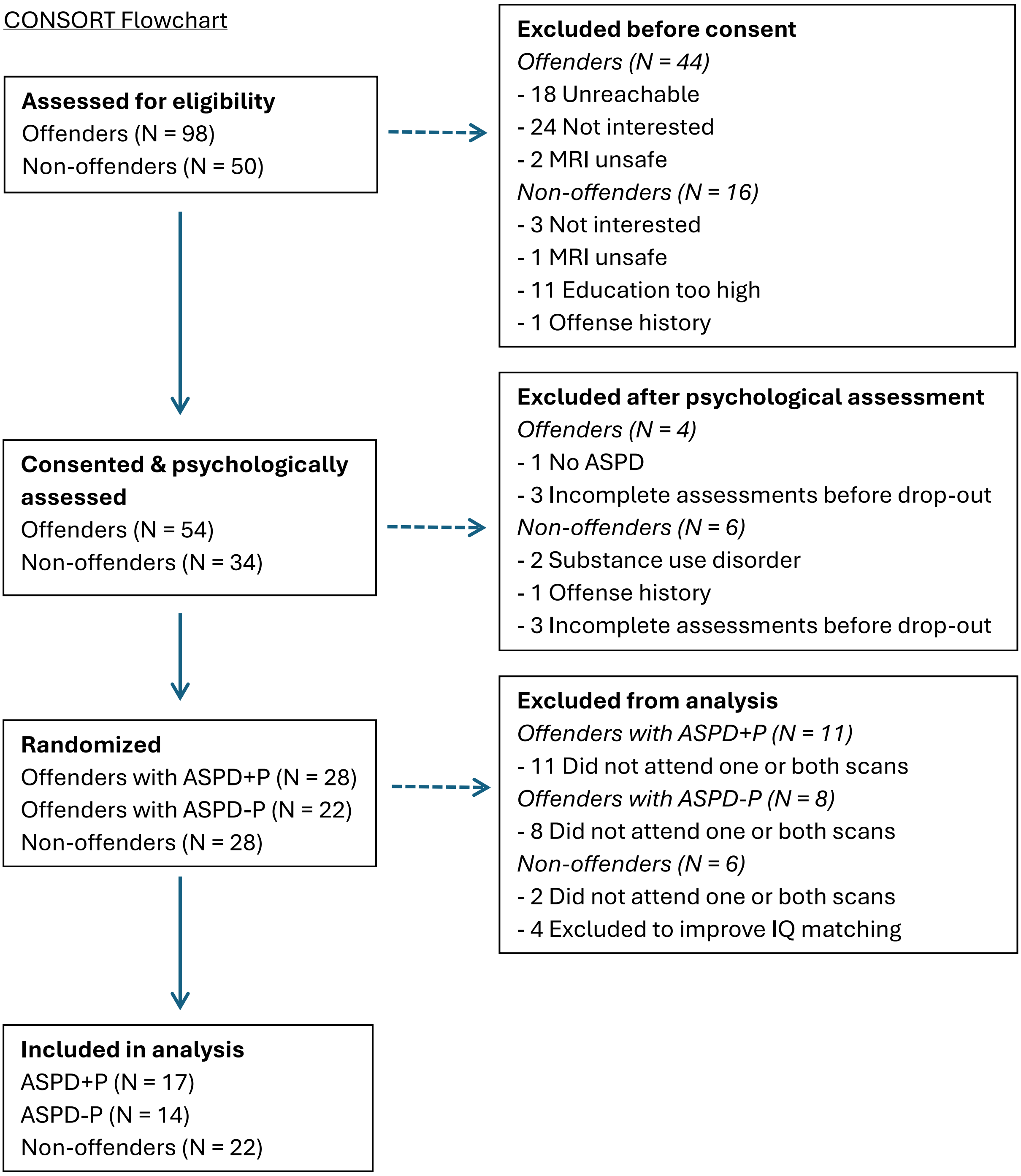

Assessment procedure

Supplementary figure 1 shows the schedule and protocol of a typical scanning day appointment. The timing for the imaging protocol was largely adhered to, though in 2 ASPD+P participants, the task-based fMRI scans were omitted due to time pressure for both placebo and oxytocin scans, bringing the ASL scan closer in time to the spray administration. The starting time (+/- 10 minutes) was also largely adhered to, however, it shifted slightly for some participants due to scanner or individual availability changes, with 1 NO participant starting the oxytocin imaging protocol at 14:19 and another at 12:38 (and placebo at normal schedule), 1 ASPD-P participant starting the placebo imaging protocol at 09:53 and another at 12:37 (and oxytocin at normal schedule), 1 ASPD-P participant starting

the oxytocin imaging protocol at 12:52 (and placebo at normal schedule), 1 ASPD+P participant starting the placebo imaging protocol at 15:38 and the oxytocin imaging protocol at 16:20, another starting the placebo imaging protocol at 16:38 and the oxytocin imaging protocol at 12:38, and a third starting the placebo imaging protocol at 09:52 and the oxytocin imaging protocol at 09:01 (the latter two being the participants that also skipped the task-based fMRI scans). Differences in timing since spray administration were accounted for across analyses, though within-subject differences in start time were not.

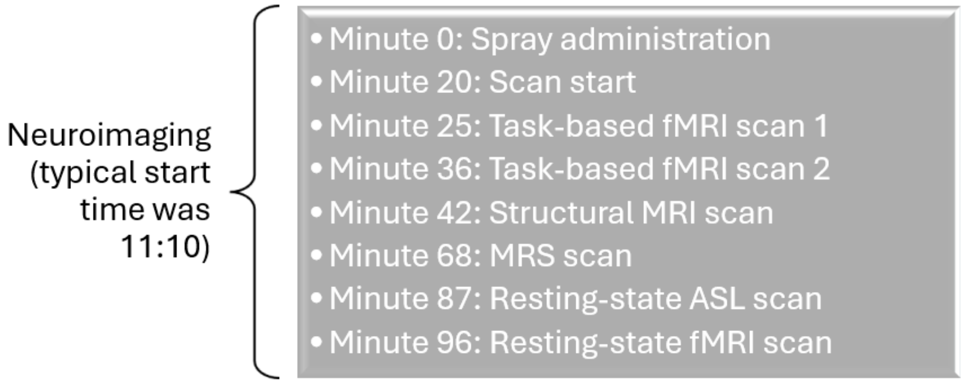

Supplementary figure 1. Overview of the schedule and planned imaging protocol for scanning days.

Further information about study procedure

As the original focus of the overarching project protocol was on task-based functional MRI, the project was designed to achieve a power of 80% with a type I error rate of $\alpha < 0.05$, significant on a single voxel level after accounting for multiple comparisons [1]. According to an a priori power analysis, this required 24 participants per group (total N = 72). Unfortunately, due to the Covid-19 pandemic, it was not possible to reach this number of participants with complete attendance. As outlined in the CONSORT flow chart, after exclusions at different stages of the study due to different reasons, the final sample included in this current study was 14-22 participants per group (total N = 53). However, two methodological studies [2,3] have shown that between-group, within-subject crossover designs require at least 7-17 participants per group to detect at least 15% change in rCBF between groups and/or after pharmacological challenge. Therefore, our study was sufficiently powered to detect a 15% within-subject change and between-group difference (effect size).

The participants who consented and completed the psychological assessments were randomly and blindly allocated to receive placebo or intranasal oxytocin on their first appointment, and the alternative on their second appointment. The nose spray bottles looked identical and all ingredients were the same, except that the oxytocin spray contained the active oxytocin ingredient (Syntocinon, Novartis, Switzerland). Staff at the Maudsley pharmacy created a restricted counter-balanced computer-generated randomization sequence with permuted blocks of six via randomizer.org. After generating this randomization sequence, they also enrolled and assigned participants to the specific treatment type for each session. Therefore, both the researchers and the participants were blind to the treatment allocation type and order.

Bayesian linear mixed models

As a post hoc assessment of non-significant results in the ROI analyses, we used R to conduct Bayesian linear mixed models, to compare these to the corresponding null models, and to

calculate the Bayes Factor (BF_{01}) which provides insight into the strength of the evidence for the null hypothesis. Here is an example of the code for the right amygdala:

```
RHAmyg.fullmodel <- brm(Median ~ (Group*Substance) +
GlobalMedian_meancentred + Age_meancentred +
MinutesSinceFinalPuff_meancentred + (1|OXYASP_ID), data=RHAmyg.data,
family = gaussian(), prior = c(set_prior("normal(0,10)", class =
"b")), iter = 4000, warmup = 1000, chains = 4, cores = 4, control =
list(adapt_delta = 0.95), save_pars = save_pars(all = TRUE))
```

```
RHAmyg.nullmodel <- brm(Median ~ GlobalMedian_meancentred +
Age_meancentred + MinutesSinceFinalPuff_meancentred + (1 |
OXYASP_ID), data = RHAmyg.data, family = gaussian(), iter = 4000,
warmup = 1000, chains = 4, cores = 4, control = list(adapt_delta =
0.95), save_pars = save_pars(all = TRUE))
```

```
RHAmygBF <- bayes_factor(RHAmyg.fullmodel, RHAmyg.nullmodel)
```

```
RHAmygBF01 <- 1 / RHAmygBF$bf
```

Supplementary results

Comorbid personality disorders in the ASPD participants

The below table outlines a breakdown of which personality disorders within each cluster participants in each group additionally met criteria for, according to the SCID-5-PD.

Demographic	ASPD+P (N = 17)	ASPD-P (N = 14)	NO (N = 22)	Group comparison	Post hoc tests (p-values)		
**PD other than ASPD, N (%)**							
**Cluster A**	5 (29%)	0 (0)	.	4.91, p = 0.05 †	.	.	.
Paranoid	5	0					
Schizoid	1	0					
Schizotypal	0	0					
**Cluster B**	8 (47%)	2 (14%)	.	3.77, p = 0.07 †	.	.	.
Borderline	4	1					
Histrionic	1	0					
Narcissistic	3	1					
**Cluster C**	1 (6%)	2 (14%)	.	0.62, p = 0.58 †	.	.	.
Avoidant	0	0					
Dependent	1	0					
Obsessive-compulsive	0	2					

Supplementary table 1. Breakdown of comorbid personality disorder diagnoses. †Fisher's exact test not significant, so no post-hoc testing completed.

Global median CBF

There was no significant main effect of group ($F(2, 48) = 0.53$, $p = .59$, $\eta_p^2 = .02$), treatment ($F(1, 48) = 1.23$, $p = .27$, $\eta_p^2 = .03$), or group by treatment interaction effect ($F(2, 48) = 1.93$, $p = .16$, $\eta_p^2 = .07$) on global median CBF (supplementary table 1).

Global Median CBF	ASPD+P	ASPD-P	NO	Main effect of group	Main effect of treatment	Group x treatment
**PL, mean (SD)**	47.59 (9.53)	46.07 (11.06)	44.77 (7.02)	F(2, 48) = 0.53, p = .59	F(1, 48) = 1.23, p = .27	F(2, 48) = 1.93, p = .16
**OT, mean (SD)**	46.35 (7.93)	41.50 (7.74)	46.36 (8.45)			

Supplementary table 2. Global median CBF mean and standard deviation (SD).

ROI analysis

The boot-strapped linear mixed models revealed no significant group, treatment, or group by treatment interaction effect on rCBF in the amygdala or the anterior insula after FDR correction for multiple comparisons (supplementary table 2). Prior to correction, there was a significant treatment effect in the left insula (uncorrected p = 0.02). The covariate of no-interest global median CBF had a significant effect on all areas, and the covariate of no-interest age had a significant effect on right and left amygdala. Minutes since dose did not have a significant effect.

Effect	Test statistic	FDR-corrected p	Effect size (μ_p^2)
**Right amygdala**			
Group	$F_{(2, 47.61)} = 1.72$	0.39	0.07
Treatment	$F_{(1, 49.06)} < 0.01$	0.98	< 0.001
Group x treatment	$F_{(2, 49.70)} = 0.90$	0.74	0.03
Global median CBF	$F_{(1, 96.79)} = 538.31$	<0.001	0.85
Age	$F_{(1, 48.26)} = 6.27$	0.04	0.10
Minutes since dose	$F_{(1, 90.47)} = 0.83$	0.48	0.01
**Left amygdala**			
Group	$F_{(2, 48.61)} = 1.55$	0.39	0.06
Treatment	$F_{(1, 49.77)} = 0.31$	0.77	0.01
Group x treatment	$F_{(2, 50.41)} = 1.72$	0.74	0.06
Global median CBF	$F_{(1, 93.81)} = 478.55$	<0.001	0.84
Age	$F_{(1, 49.28)} = 8.75$	0.02	0.15
Minutes since dose	$F_{(1, 95.43)} = 0.05$	0.83	< 0.001
**Right insula**			
Group	$F_{(2, 47.81)} = 1.33$	0.39	0.05
Treatment	$F_{(1, 48.11)} = 0.68$	0.77	0.01
Group x treatment	$F_{(2, 48.62)} < 0.01$	0.99	< 0.001
Global median CBF	$F_{(1, 73.75)} = 569.89$	<0.001	0.89
Age	$F_{(1, 48.39)} = 0.07$	0.80	0.002
Minutes since dose	$F_{(1, 88.97)} = 1.96$	0.35	0.02
**Left insula**			
Group	$F_{(2, 47.88)} = 1.06$	0.39	0.04
Treatment	$F_{(1, 48.77)} = 5.34$	0.08*	0.10
Group x treatment	$F_{(2, 49.38)} = 0.61$	0.74	0.02
Global median CBF	$F_{(1, 88.33)} = 569.70$	<0.001	0.87
Age	$F_{(1, 48.55)} = 0.35$	0.80	0.007
Minutes since dose	$F_{(1, 97.00)} = 3.94$	0.21	0.04

Supplementary table 3. ROI analysis of rCBF in amygdala and anterior insula. * Uncorrected p = 0.02.

To examine the non-significant results further, we conducted Bayesian linear mixed models. The full model, which included the fixed effects of group, treatment, and their interaction, alongside covariates (global median CBF, age, and minutes since dose) as well as the random effect of subject was compared to the null model, which only included the covariates and the random effect of subject. For the right amygdala, Bayes Factor (BF_{01}) was 780772148, for the left amygdala, BF_{01} = 249713017, for the right insula, BF_{01} = 261256027, and for the left insula, BF_{01} = 29610173. Across all four ROIs, this suggests that there is compelling evidence in favour of the null hypothesis, indicating that group, treatment, or their interaction do not significantly influence median rCBF after adjusting for the covariates. This validates the results from the frequentist linear mixed model.

Effect of substance use

Recent substance use was measured with a multi-panel urine drug test which tested for THC, cocaine, opioid, PCP, amphetamine, methamphetamine, barbiturates, benzodiazepine, and methadone use. The most frequently used substance across participants were cannabis and cocaine. Supplementary table 4 provides a granular level of detail, comparing the number of

positive drug tests for each of these individual substances between the three groups on each day of scanning. After adjusting for multiple comparisons (Sidak correction), this confirms that the rate of substance use according to urine drug testing only differed significantly across groups for cocaine, driven by a significant higher rate of use in the ASPD+P group as compared to the non-offending group.

Sensitivity analyses were conducted to assess the effect of recent substance use as indicated by a positive urine drug test from the day of each MRI scan. Specifically, this binary variable was included as an additional covariate (alongside age, global rCBF, and time since administration) in the post-hoc tests that were used to interpret the main effect F-contrasts.

The results remained largely the same as without including this variable as covariate. Only the ASPD-P vs NO comparison in cluster 4 lost significance. All other results remained the same or became more significant.

	ASPD+P	ASPD-P	NO	Main statistic (Fisher's exact test)	ASPD+P vs NO	ASPD-P vs NO	ASPD+P vs ASPD-P
**Placebo scan**							
**THC**	8 (47%)	3 (21%)	5 (23%)	5.31, p = .18	.	.	.
**Cocaine**	5 (29%)	1 (7%)	0 (0%)	9.77, p = .007	p = .01	p = .14	p = .19
**Opioid**	2 (12%)	0 (0%)	1 (4.5%)	3.97, p = .34	.	.	.
**PCP**	0 (0%)	0 (0%)	1 (4.5%)	3.80, p = .56	.	.	.
**Amphetamine**	1 (6%)	0 (0%)	0 (0%)	4.32, p = .24	.	.	.
**Methamphetamine**	1 (6%)	0 (0%)	0 (0%)	4.31, p = .24	.	.	.
**Barbiturates**	0 (0%)	1 (7%)	0 (0%)	2.37, p = .26	.	.	.
**Benzodiazepine**	4 (24%)	0 (0%)	0 (0%)	8.90, p = .008	p = .03 $^{ns◊}$	p = .39	p = .11
**Methadone**	0 (0%)	0 (0%)	0 (0%)	n/a	.	.	.
**Oxytocin scan**							
**THC**	9 (53%)	3 (21%)	5 (23%)	4.64, p = .11	.	.	.
**Cocaine**	5 (29%)	2 (14%)	0 (0%)	7.29, p = .01	p = .01	p = .14	p = .41
**Opioid**	2 (12%)	2 (14)	0 (0%)	3.38, p = .16	.	.	.
**PCP**	0 (0%)	0 (0%)	1 (4.5%)	1.47, p = 1.00	.	.	.
**Amphetamine**	1 (6%)	0 (0%)	0 (0%)	1.98, p = .59	.	.	.
**Methamphetamine**	1 (6%)	0 (0%)	0 (0%)	1.98, p = .59	.	.	.
**Barbiturates**	0 (0%)	0 (0%)	0 (0%)	n/a	.	.	.
**Benzodiazepine**	4 (24%)	1 (7%)	0 (0%)	5.56, p = .04	p = .03 $^{ns◊}$	p = .39	p = .34
**Methadone**	0 (0%)	1 (7%)	0 (0%)	2.37, p = .26	.	.	.

Supplementary table 4. Drug use as measured on urine drug tests conducted on the day of each scan. Main test statistic was a Fisher's exact test, and where significant, a Fisher's exact test was conducted as a post-hoc. These post-hoc tests were corrected for 3 group comparisons using Sidak correction, whereby the adjusted α threshold was p = 0.017. Therefore, 2 post-hoc tests did not survive correction for multiple comparisons, as indicated by the superscript ns◊.

1. Desmond JE, Glover GH. Estimating sample size in functional MRI (fMRI) neuroimaging studies: Statistical power analyses. J Neurosci Methods. 2002;118:115–128.

2. Murphy K, Harris AD, Diukova A, Evans CJ, Lythgoe DJ, Zelaya F, et al. Pulsed arterial spin labeling perfusion imaging at 3 T: estimating the number of subjects required in common designs of clinical trials. Magn Reson Imaging. 2011;29:1382–1389.

3. Mutsaerts HJMM, Steketee RME, Heijtel DFR, Kuijer JPA, van Osch MJP, Majoie CBLM, et al. Reproducibility of pharmacological ASL using sequences from different vendors: implications for multicenter drug studies. Magn Reson Mater Physics, Biol Med. 2015;28:427–436.
